# *Debaryomyces hansenii* Is a Real Tool to Improve a Diversity of Characteristics in Sausages and Dry-Meat Products

**DOI:** 10.3390/microorganisms9071512

**Published:** 2021-07-15

**Authors:** Laura Ramos-Moreno, Francisco Ruiz-Pérez, Elisa Rodríguez-Castro, José Ramos

**Affiliations:** Agriculture Microbiology Research Group, Department of Agricultural Chemistry, Edaphology and Microbiology, University of Córdoba, E-14071 Córdoba, Spain; lauraramosm89@gmail.com (L.R.-M.); b42rupef@uco.es (F.R.-P.); q72rocae@uco.es (E.R.-C.)

**Keywords:** *Debaryomyces hansenii*, yeast, sausages, dry-meat products, starter cultures

## Abstract

*Debaryomyces hansenii* yeast represents a promising target for basic and applied biotechnological research It is known that *D. hansenii* is abundant in sausages and dry-meat products, but information regarding its contribution to their characteristics is blurry and contradictory. The main goal in this review was to define the biological contribution of *D. hansenii* to the final features of these products. Depending on multiple factors, *D. hansenii* may affect diverse physicochemical characteristics of meat products. However, there is general agreement about the significant generation of volatile and aromatic compounds caused by the metabolic activities of this yeast, which consequently provide a tendency for improved consumer acceptance. We also summarize current evidence highlighting that it is not possible to predict what the results would be after the inoculation of a meat product with a selected *D. hansenii* strain without a pivotal previous study. The use of *D. hansenii* as a biocontrol agent and to manufacture new meat products by decreasing preservatives are examples of exploring research lines that will complement current knowledge and contribute to prepare new and more ecological products.

## 1. Introduction

The microbiome of sausages and dry-meat products is an important factor contributing to the characteristics of these foods. Lactic acid bacteria and some micrococci and staphylococci are predominant groups of prokaryotic microorganisms found in fermented sausages that play an important role in the fermentative process and ripening. In fact, the role of bacteria in the manufacture of meat products is quite well known compared with that of yeast [1,2,3,4,5] since, in many cases, although several yeast have been isolated and identified their function has not yet been studied. Multiple reports on the diversity of yeast populations in sausages and other dry-meat products have indicated that *Debaryomyces hansenii* is one of the most abundant and commonly found species in these products [6,7,8,9,10]. However, there is not complete agreement about the real importance of *D. hansenii* in the ripening and final characteristics of these foods, and controversial results are available in literature concerning its effects on the quality and/or sensory characteristics of fermented meat products. Therefore, the influence of *D. hansenii* on the quality and/or sensory characteristics of fermented meat products has not been completely defined. 

In general, the contribution of yeast populations to flavour and aroma in meat products is usually related to their ability to ferment sugars and to degrade amino acids, consequently producing ethanol, acetaldehyde, or ethyl acetate, as well as a variety of other volatile compounds [11]. Moreover, yeast also metabolizes lactic acid produced by lactic bacteria, thus affecting the organoleptic characteristics of the product. However, it is important to note that other yeast genera such as *Rhodotorula, Yarrowia, Candida*, *Trichosporon, Citeromyces*, *Kazachstania*, or *Wickerhamomyces* are commonly present in meat products and that flavour and aroma changes cannot be solely attributed to *Debaryomyces* [9,12,13,14,15,16]. In addition, a pertinent question is whether changes in the products due to the yeast populations can be finally perceived by the consumer and affect the overall sensory characteristics of the products.

Published information on the functions of *Debaryomyces* is confusing, blurry, and sometimes even contradictory, probably due to the fact that *D. hansenii* is a highly heterogeneous species with wide phenotypic differences and is sometimes miss-identified [17]. Naturally occurring strains are supposed to improve products, however there is no general conclusion regarding the consequences of the inoculation of meat products with *D. hansenii*. Therefore, it is difficult to predict positive effects on the final products. 

The main issue addressed in this work is to define the biological importance of *D. hansenii* in the preparation of sausages and dry-meat products independently of the specific manufacturing characteristics of the different foods. The specific objectives of this review are to: (i) contribute to unravelling and deciphering the real importance of the presence of *D. hansenii* in meat products; and (ii) to analyse the expectations created about the use of *D. hansenii* in other novel aspects of research, such as the reduction in the use of preservatives or its potential role as a biocontrol agent.

## 2. *Debaryomyces hansenii* as a Non-Conventional Yeast of Biotechnological Importance

*Debaryomyces hansenii* is a hemiascomycetous yeast of undoubted biotechnological importance [18]. It is a heterogeneous yeast species able to grow under extreme conditions, such as high salt or relatively alkaline pH levels. This yeast has high respiratory and low fermentative activity [19]. It ferments with important variations, depending on the strain and growth conditions used [20,21]. Briefly, some examples of its beneficial effects are the production of xylitol [22], lipases, and exopeptidases important in the food industry, and of thermophilic β-glucosidases essential to produce fuel alcohol [18]. Moreover, it is considered as a model of eukaryotic microorganism for the study of osmotic adaptations and salt tolerance. It can grow in the presence of different carbon sources and produces killer toxins [17,18]. Very interestingly, *D. hansenii* seems to have the highest coding capacity amongst yeasts [23]. This microorganism colonizes a great diversity of specific microhabitats, indicating the existence of yeast subpopulations adapted to such habitats and showing chromosomal polymorphism. In this way, it has been reported that specific probes can be useful to investigate biodiversity within *D. hansenii* [24].

Despite the potential of this yeast, different circumstances have hindered its habitual scientific and biotechnological use and have prevented the further development of its industrial applications [17,18]. Linked to its heterogeneous diversity, there is also some confusion regarding its negative effects to human health. Since the early 1980s *D. hansenii* has been assimilated with other species as *Candida famata*, which has been considered its clinically anamorph. These identifications were mainly based on inaccurate phenotypic characteristics that are now being questioned. As a consequence of the difficulties related to separating *D. hansenii* from closely related yeasts, a diversity of biochemical and molecular approaches are currently in use to clearly identify this species [24,25,26,27,28,29]. Once again, several reports using molecular tools to discriminate between species have described the misidentification of *Pichia guilliermondii* and others as *D. hansenii/C. famata* [30,31,32]. Furthermore, some of the previously identified *C. famata* strains have now been included in other *Debaryomyces*-related species such as *D. fabryi* or *Candida flareri* [33]. Although *D. hansenii* is closely related to *Candida* and some authors have reported *D. hansenii* as an emergent pathogen, so far, no clear clinical significance has been attributed to this yeast and therefore it is not responsible for generalized or common health problems, and we can conclude that it is not relevant from a clinical point of view. Altogether, these studies suggest that D. *hansenii* may be a very rare (or even non-existent) human pathogen.

The lack of user-friendly molecular tools to manipulate this yeast has slowed the research on *D. hansenii*; in addition, the fact that *D. hansenii* is not clinically significant could also be considered as a factor for its research disinvestment [30]. For example, the number of well-characterized genes/proteins from *D. hansenii* is still very low and limited to a few families. Finally, it is worth noting that *D. hansenii* belongs to the ambiguous CUG decoding group of yeasts, in which CUG codon can be ambiguously translated, mainly as serine but also as leucine, although in a minor percentage [34]. 

## 3. *Debaryomyces hansenii* Is One of the Most Abundant Yeasts in Sausages and Dry-Meat Products

The usual approach to the study of the microbiome present in dry-meat food and sausages is the isolation and identification of the main populations present in these products. In this way, yeast isolates have been obtained from many different meat products during the last decades. Although their functions and effects in meat were not always defined in detail, there is no doubt that previous studies demonstrate that *Debaryomyces hansenii* is frequently and abundantly found in sausages and dry-meat products [6,7,8,9,29].

The identification of *D. hansenii* as the most common yeast in salami was made as early as 1954 [35]. Since then, and especially in the last two decades, the presence of multiple strains of *D. hansenii* in sausages and dry-meat products has been reported. Table 1 summarizes some of these products and their origins. Many countries where sausages and dry-meat foods have been studied belong to the Mediterranean area [8,36,37,38,39,40,41], but dry-cured meat or sausages are prepared and consumed by millions of people worldwide and also in other European countries such as Denmark [42], Norway [43], Austria [7], the United Kingdom [6], and Portugal [44]. Even in places such as Argentina [45] or China [46,47], among many others, meat products are manufactured in which it is possible to find *D. hansenii* yeasts, supposedly contributing to the final organoleptic characteristics of the food.

As already mentioned, the very diverse metabolic discrepancies of *D. hansenii* have been observed in naturally occurring strains isolated from the same meat products. For example, differences in the capacity to assimilate xylose [8,59], in the urease activity [8] or in specific lipolytic and proteolytic enzymatic activities [29], have been reported. However, several groups have obtained similar qualitative results coincident when describing some important physicochemical characteristics in *D. hansenii* isolates from different origins. For example, a high resistance to NaCl or the sensitivity to relatively high temperatures (37 °C) have been usually observed in *D. hansenii* strains found in sausages from Italy or Spain [8,29].

From the different studies reporting on yeast populations in sausages and dry-cured meat products, it can be deduced that even the behaviour of the yeast populations during the ripening of the dry-meat products do not seem to be homogeneous. Thus, while a microbiological study of Greek salami reported that yeasts did not significantly increase in the ripened product (after 28 days) [60], the yeast counts in sausages of southern Italy significantly increased during the first days of fermentation, subsequently remaining constant or even decreasing [8]. As a final example and in the case of dry-cured Iberian ham, higher counts of yeast were reached at post-salting or at drying periods and then the number decreased after several months in a cellar [37]. In any case, and independently of the manufacturing process, naturally occurring yeasts are usually found in high numbers indicating that these microorganisms may play an important role in the maturation process [50]. Therefore, it is generally accepted that the yeast populations, including *D. hansenii*, present in artisanal-style products contribute to flavour, colour, and texture development during the ripening of these products. However, the presence of competing bacteria, mostly lactic acid bacteria, and other organisms has made it difficult to reach solid conclusions about the functions and contributions of specific species. That is why most of these studies have focussed their attention on quantifying, identifying, or partially characterizing yeast species during the manufacturing process and/or the maturation period [8,13,15,29]. Very recently some research groups have used “omic” and multidisciplinary approaches to analyse the microorganisms during processing or ripening in traditional Chinese products [46,47]. Bacteria and fungi were identified, for example during panxian ham processing, a traditional Chinese dry-cured ham, and it was concluded that *Staphylococcus*, *Chromohalobacter*, and *Debaryomyces* promoted the production of amino and fatty acids [47], but once again the presence of naturally occurring complex communities made it difficult to reach more specific conclusions.

In summary, *D. hansenii* is naturally present in significant numbers in many different dry-meat products and sausages, however it is difficult to isolate its role in final organoleptic characteristics mainly due to the presence of other populations of microorganisms that also influence the maturation of the product. Therefore, the inoculation of high amounts of *D. hansenii* as a sole starter is the next in an attempt to unravel the effects this yeast has on dry-meat products by comparison with control batches containing only their natural microbiome.

## 4. *Debaryomyces hansenii* as a Starter Culture: Effects on the Final Characteristics of Meat Products

How *Debaryomyces hansenii* activity effects the organoleptic properties and appearance of sausages and dry-meat products has not been sufficiently clarified. The contributions of yeast to the final characteristics of these products can be related to its ability to ferment different sugars, to degrade peroxides and amino acids, and to its lipolytic activity [11,61,62,63]. Several research groups have approached many of these issues from different points of view and under diverse conditions. The use of *D. hansenii* as a starter culture, through the inoculation of high amounts of a specific strain, can undoubtably help to understand the role of this species during ripening and the consequences of its presence. The group of M. Flores, for example, has very actively analysed the impact of *D. hansenii* strains on meat products in the presence of low salt or by manufacturing sausages with entirely male fat [64,65,66]. It is important to keep in mind that, as mentioned below, some papers report the use of pure *D*. *hansenii* starters while some others use the yeast in combination with additional microorganisms which must be considered in order to reach conclusions [54,67,68].

We summarize below some of the main changes in meat products that have been reported to be induced by the presence of *D. hansenii.*

### 4.1. Lipolysis

*D. hansenii* shows lipolytic activity. In 1997 it was shown that a commercial starter of this yeast could hydrolyse a natural fatty substrate like pork fat and release fatty acids. In this case, lipolysis caused by *D. hansenii* was not affected by NaCl (most probably due to the salt-tolerant character of this yeast) and it was still significant at pH 4.7, indicating that this commercially available starter culture may hydrolyse pork fat during the processing of fermented meat products [69].

Later, our group has shown that the lipase activity of several strains isolated from Iberian dry-meat products greatly differed among strains, furthermore it was always higher than the corresponding activity in a control laboratory strain (CBS767) [29]. 

More recently, we used a selected terroir strain isolated from pork loin, *D. hansenii* Lr1, to inoculate different amounts of yeast either directly onto the meat surface or onto the collagen casing in which each loin piece was stuffed to make the product. Possible changes in the lipid profile of the loins were determined. In all cases, including control samples, loins contained a very low percentage of polyunsaturated fatty acids (PUFAs) and the most abundant were monounsaturated fatty acids (MUFAs). On the other hand, inoculation with the Lr1 strain did not significantly change the fatty acid profiles of any of the treatments applied. Within all the samples, oleic acid (C18:1) constituted around 50%, palmitic acid (C16:0) around 26%, and stearic acid (C18:0) around 10–12% of the total fatty acids. Clear differences could not be found related to the different treatments and only slight, although consistent, effects on the percentage of some fatty acids were observed as, for example, all the treatments showed a decrease in the percentage of t-oleic acid (elaidic acid, C18:1 n9t) when compared to the control samples. In summary, it was shown that inoculation with the *D. hansenii* Lr1 strain did not significantly change the global lipid profile of the loins. Only the amounts of some fatty acids were affected, however changes in the total amounts of saturated fatty acids (SFA), MUFAs, and PUFAs were not significant [58].

However, a different study showed that the generation of free fatty acids during sausage fermentation is affected by yeast inoculation. In this case, the *D. hansenii* P2 strain, previously isolated from naturally fermented sausages “salchichón de Requena [54,70], was inoculated in sausages manufactured with boar back fat or with gilt back fat. It was demonstrated that inoculated sausages had a higher degree of lipolysis and that this was strongly dependent on the ripening time and the conditions. After 63 days of ripening, an increase in the content of free MUFAs, PUFAs, and total free fatty acids was measured in the boar sausages while, in general, lower, or even no significant differences, were observed after 43 days of ripening or when the sausages were manufactured using gilt back fat instead of boar back fat. The authors concluded that environmental conditions affect lipase activity or lipase expression genes, potentially explaining differences between strains or products [66]. 

In summary, experimental evidence suggests that the inoculation of dry-meat products with *D. hansenii* can affect their lipid profile but that this does not seem to be pivotal to the outcome, and, in any case, it depends on multiple factors that may mask and/or alter the specific effect of the yeast.

### 4.2. Volatile Compounds

A generally accepted idea is that the generation of volatile compounds by *D. hansenii* is one of the most important contributions to the ripening process in dry-meat products. It is usually found that the introduction of *D. hansenii* as a starter culture affects volatile and aromatic compound generation. Among them, esters are essential contributors to the aroma of meat products due to their low detection threshold and sensory notes. The production of these compounds represents a complex scenario and it is strongly dependent on the different isolates and strains and even on the amount of yeast used [54,55]. In fact, production of volatile sulphur compounds from sulphur amino acids greatly varied among *D. hansenii* strains isolated from different food sources and the generation of, at least, some of these compounds could result from yeast metabolism [71].

Several groups have used starter cultures containing *Debaryomyces* in combination with other different microorganisms. For example, when dry-fermented sausages were inoculated with *Debaryomyces* spp. plus lactic acid bacteria and staphylococci, it was concluded that the use of this combination of microorganisms had a positive effect on the final flavour and sensory qualities of the product influenced by the generation of ethyl esters [72]. Importantly, the authors found that the amount of yeast used in the starter culture must be optimised, since too many yeasts may mask some positive effects. More recently, a similar approach was followed to study the effects of different starter cultures on volatile compounds of dry-cured foal sausages. In this case, three different starters were used and while two of them contained only bacteria, the third one was prepared with bacteria (*Lactobacillus sakei, Staphylococcus carnosus*, and *Staphylococcus xylosus*) and yeast (*D. hansenii*). Significantly different effects on the volatile compounds or acid taste were found among the different batches, with the batch containing *D. hansenii* showing a high flavour intensity and high levels of compounds derived from carbohydrate fermentation and amino acid catabolism [73]. The use of mixed starters containing only fungi has been less frequent. When *Penicillium chrysogenum* and *D. hansenii* were inoculated on dry-cured ham they did not remarkably alter the volatile compound profile. Only lower levels for some of the main odour-active volatile compounds were measured but they were not detected by a panel of experts [48].

Evidently, conclusions about the specific contributions of *D. hansenii* must be analysed from a global point of view, since all these experiments were performed with mixed starter cultures. Due to the difficulties surrounding the proper interpretation of the results obtained in these types of experiments, many other groups have focused on the use of pure *D. hansenii* starters [42,49,50,58].

**Figure 1:**
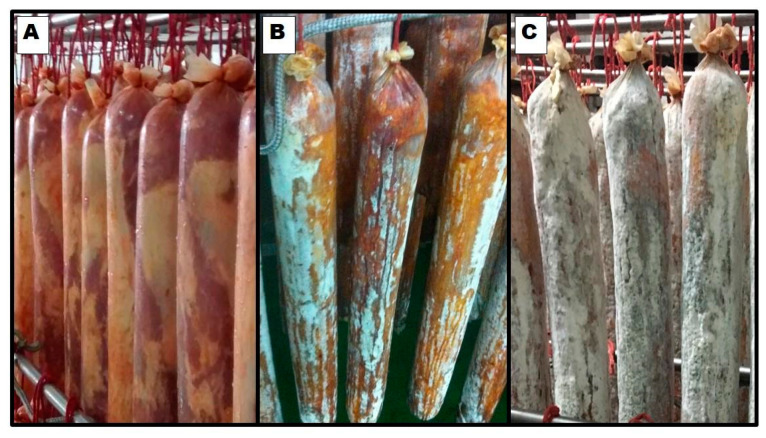
Implantation of inoculated *Debaryomyces hansenii* yeasts on the surface of pork loins after 0 (**A**), 10 (**B**), and 30 (**C**) days of ripening.

One of the first realistic attempts to understand the specific effect of *D. hansenii* on aroma formation was performed by Olesen and Stahnke [42] in spiced fermented sausages. It was found that *D. hansenii* had very little effect with the analysis showing only a slight difference between the inoculated sausages and the control, possibly due to the fact that the yeast died out before the ripening process ended. The main reason was that sausages were spiced with garlic, and a fungistatic test of the garlic powder added to the sausages indicated that garlic inhibited the growth of the yeast starter cultures [42]. Later studies showed that *D. hansenii* contributes to the development of the characteristic flavour of some of these dry-meat products [49,50,58]. A research conducted on the dry-fermented sausage ‘‘salchichón” and performed with different *D. hansenii* strains indicated that the inoculation of selected isolates may have a positive contribution to the volatile compound generation involved in the flavour development of this meat product. In this study, yeasts were incorporated into the batches and the mixture of each batch was stuffed into regenerated collagen casings. The tested yeast strains promoted the generating of esters, alcohols, and aldehydes, and some volatile compounds derived from lipid oxidation [50]. In addition, when the effect of *D. hansenii* on dry-cured “lacon” and Iberian cured pork loin (“lomo ibérico”) was studied, similar approaches were followed: strains were selected from native products and used as starters. Yeasts were spread on the surface of the meat product and their capacity to generate volatile compounds was determined. Figure 1 shows the proliferation of inoculated *D. hansenii* on the surface of pork loin during the ripening period. As expected, quantitative differences were found when both studies were compared. This is not surprising, since both strains and meat products were different. Nonetheless, important qualitative similarities were found. In all cases, and in agreement with previous work in “salchichón”, *D. hansenii* modified the levels of volatile and aromatic compounds by increasing esters and alcohol metabolites in comparison to the non-inoculated samples [49,58]. However, and in contrast with what was reported in the case of “salchichón”, a significant decrease in aldehydes was reported in both “lacon” and “lomo ibérico”.

In some cases, a different approach was followed, since model minced meats which did not correspond to any specific commercial meat products were used. A first attempt by Olesen and Stahnke [42] indicated that the use of *D. hansenii* as a starter culture had very little effect on the production of volatile compounds in the model minces, although, as previously mentioned, the presence of garlic affected cell viability. More recently, the ability of several *D. hansenii* strains to generate aromas in a fermented sausage model system was evaluated. The performance of seven different strains previously selected on their ability to produce aromatic compounds in a defined culture media [70] were later studied in a meat model system. The presence of each inoculated strain was confirmed in the model system and an increase in volatile compound production was observed in all cases. However, significant differences were found among strains, especially in relation to ester production which was correlated to the lipolytic activity of the strains. Sulphur production was also strongly dependent on the inoculated strain. In summary, it was concluded that: (i) the inoculated *D. hansenii* strains affected the flavour development of the meat model system; (ii) wide differences do exist among strains, although in all cases volatile compounds increased; (iii) the meat model system is useful to show the ability of *D. hansenii* strains to produce aromatic compounds; and that (iiii) it is necessary to investigate the effects of specific strains in real dry-fermented sausages [54,55].

Finally, in a more complex study and working with dry-fermented sausages manufactured with reduced salt content and several types of fats, again the production of aldehydes was reduced while the production of alcohols and esters was increased in the presence of *D. hansenii* [66].

### 4.3. Other Physicochemical and Sensory Characteristics Affected by Debaryomyces hansenii

#### 4.3.1. pH

Several studies have shown that changes occur in pH levels during the processing of meat products. The pH level is always lower at the end of the ripening period independently of the presence of yeast. Inoculation with mixed starter cultures (bacteria plus *Debaryomyces*) resulted in the higher acidification of sausages [73]. However, as previously mentioned, the specific contribution of yeast species cannot be easily deduced due to the presence of bacteria in the starter mix [73]. When *D. hansenii* was used as starter it was difficult to find a general conclusion regarding it effect on the final pH of the product, although it is possible to state that the consequences of the yeast activity did not acutely affect pH and that these consequences are strongly dependent on both the yeast strain and product. In some cases, similar pH values to the control were measured at the end of the process, while in some other studies the final pH values slightly higher or lower than non-inoculated products were determined [11,50,54,58]. In conclusion, few and variable changes in pH have been reported in yeast-inoculated meat products.

#### 4.3.2. Water Activity and Moisture

More homogeneous results were obtained by the different researchers when water activity (A_w_) or moisture was determined. It has been fully demonstrated that both parameters decrease along the ripening period in both control and inoculated samples. On the other hand, while higher final values of A_w_ were usually measured and explained as a consequence of yeast growth on the product surface [50,58,65], no significant differences in moisture were found when results in control and inoculated products were compared [11,54,65,73].

#### 4.3.3. Sodium Content

The possible effects of yeast on the sodium levels of meat products has not been frequently studied. While commercial mixed starter cultures did not affect saltiness of dry-cured foal sausages [74], only in the case of cured pork loins has an effect of *D. hansenii* inoculation on the sodium content of the product been reported [58]. Interestingly, yeast inoculation produced a significant decrease in the sodium content of the product. It is known that *D. hansenii* can accumulate high amounts of sodium from the environment without becoming intoxicated and for this reason it has been defined as a sodium-includer yeast [75,76,77]. This behaviour may explain why inoculated loins contain lower sodium amounts, a characteristic not previously reported.

#### 4.3.4. Colour

Heterogeneous results regarding the consequences of yeast use on this parameter have been reported. On the one hand, the use of *D. hansenii* to inoculate standard slow dry-cured fermented sausages did not affect colour parameters such as redness, yellowness, or lightness [54]. However, the same research group later reported that in the specific case of sausages produced with entirely male fat, yeast inoculation affected colour parameters, thus lightness values were significantly higher in the inoculated sausages after 43 days of drying [65].

Once again it is important to keep in mind that many of the effects described depend on the meat product and the manufacturing process followed.

### 4.4. Sensory Properties Affected by Debaryomyces hansenii

We have concluded so far that, on many occasions, the inoculation with *Debaryomyces hansenii* of various meat products produces significant changes in some of their physicochemical characteristics. A crucial point now is to consider whether all this translates into sensory level changes that are detectable by the consumer 

The results of the inoculation of meat products with *Debaryomyces* on their sensory characteristics are controversial. While some studies have reported significant effects [67,72], other papers showed an absence of sensory effects [42,78]. The authors concluded that results can be affected by the selected strain, the quantity of the inoculated yeast, the type of sausage, the manufacturing process, and even the possible presence of other starters.

#### 4.4.1. Mixed Starters

The combination of non-toxigenic moulds and yeasts as starters has been rarely approached. When selected *Penicillium chrysogenum* and *Debaryomyces hansenii* strains were inoculated on dry-cured ham, lower levels of some of the main odour-active volatile compounds were measured but not detected by panellists, and a better overall acceptability for inoculated hams was observed and was attributed to their improved texture [48].

In a detailed study, different amounts of *Debaryomyces* spp. in combination with bacterial starter cultures were used. A final yeast population of 5 × 10^6^ or 15 × 10^6^ cfu/g were inoculated in dry-fermented sausages. The sensory analysis showed that the panel significantly preferred the batch with the lower amounts of yeast, but no differences between the control and the 15 × 10^6^ cfu/g batches of sausages were found. It was concluded that the yeasts had a positive effect on the sensory characteristics, probably due to the production of ethyl esters that contribute to the proper sausage aroma. It was also concluded that the amount of yeasts present was important, as larger amounts produce a higher generation of acids that may mask the positive effects of *Debaryomyces* [72]. 

In a different research, once again *D. hansenii* isolates from natural fermented sausages in combination with bacterial starter cultures were inoculated in slow fermented sausages. In this case, two different *D. hansenii* strains at a final concentration of 1 × 10^6^ cfu/g were studied. Although significant differences were measured for the production of volatile compounds, these products were not appreciated by the consumer panel, which was related to the interference of the bacterial starter used in the ripening process [54]. Moreover, the results also pointed out the importance of standardizing the yeast concentration used in each experiment.

#### 4.4.2. Pure Yeast Starters

As previously mentioned, the main advantage of using only pure yeasts and not mixed populations of microorganisms as starters is that this provides the possibility of formulating more solid conclusions on the role of a specific strain. 

Iucci et al. [67] examined the sensory properties of dry-fermented sausages inoculated with selected *D. hansenii* D9 or *Yarrowia lipolytica* Y16 strains. The sensory analysis showed that the sausages inoculated with *D. hansenii* were generally preferred by the panellists, while the inoculation with *Y. lipolytica* rendered products less appreciated than the control, non-inoculated ones by the group of experts. It is worth mentioning that the degree of mincing affected the sensory properties of the sausages and the necessity of identifying tailor-made yeast starters for each type of sausage and production process was stressed. Later, different biotypes of *D. hansenii* were used to investigate the influence of *D. hansenii* used as single starter culture on the volatile compound generation of dry-fermented sausage “salchichón” and, again, differences between strains were reported [50].

In the case of pork loin sample surfaces inoculated with two different amounts of a selected terroir *D. hansenii* strain, the consumer sensory panel did not detect any changes in texture among treatments. However, significant differences were found in all the other attributes evaluated when compared to the control samples. In general, all treatments improved in a similar way the acceptability of the product in comparison to the control, including the aspect of the samples. Moreover, a positive effect with respect to aroma and flavour, and a tendency to higher global consumer acceptability for samples inoculated with higher dose of yeast, was observed. Relevant also was the improvement in the salty taste that the inoculated yeast produced, most probably was due to the sodium-includer characteristics of *D. hansenii* [58].

Table 2 recapitulates some of the main factors conditioning the effect of *D. hansenii* on the final characteristics of sausages and dry-meat products. In summary, most of the studies highlight the difficulty of obtaining generalized and definitive conclusions on the effects of yeast inoculation on the sensory characteristics of fermented products without a previous and specific study. In some cases, a better consumer acceptance is reported and in some no clear differences are found. Once more, the strains, the amount of yeast, the type of product, or specific manufacturing conditions are crucial to define the possible consequences of the use of a *D. hansenii* starter.

## 5. Other *Debaryomyces hansenii* Functions Beyond Just Improving Overall Characteristics of Sausages and Dry-Meat Products

The meat products mentioned in this review are enormously diverse, both in their origins and in their preparation. However, the steps followed to unravel everything related to the possible role of yeasts in the characteristics of these products are usually highly standardized: first the isolation and identification of yeast genera, followed by a selection of those with the most interesting potential and, finally, the inoculation as a starter and subsequent physicochemical analysis to identify changes in the product.

Much more recently, novel research lines are being opened in order to discover possible additional functions of the yeast population. For example, (i) the inoculation of fermented sausages with *Debaryomyces hansenii* has been shown to contribute to masking unwanted fat odours due to its lipolytic activities [65,66]; (ii) the potential use of mixed starters containing *D. hansenii* to reduce the risk of meat mutagens and biogenic amines in fermented sausages is being studied [79,80]; or (iii) the efficacy of *Debaryomyces hansenii* for controlling *Listeria monocytogenes* has been evaluated in dry-cured ham [81]. In addition, the possible contributions of *D. hansenii* both to reducing preservatives and antimicrobial activities, especially against pathogens, are two research lines of great interest and potential.

### 5.1. Counteracting the Negative Impact of Preservatives

The use of nitrite and nitrate as curing agents in dry-fermented sausages has important technological functions. Nitrite has antioxidant and antimicrobial activities, of which its activity against the pathogen *Clostridium botulinum* and its control of toxin production are the most important. Furthermore, nitrite facilitates the generation and stabilization of the typical colour of the product and enhances the cured flavour [82,83].

An important and actual trend is to reduce the use of nitrite and nitrate in meat products due to the controversy about the adequate amounts of nitrite used as additive in meat products [84,85]. However, the consequences of using reduced levels of nitrate and nitrite mixtures on the aroma profile of dry-cured meat products have been scarcely studied. The inoculation of yeasts such as *D. hansenii*, which have the potential to generate desirable aromas in dry-fermented sausages, is a suitable and promising alternative. The effect of the inoculation of a *D. hansenii* strain on dry-fermented sausages manufactured with reduced ingoing amounts of nitrite and nitrate has been very recently evaluated. In a first study, it was reported that different D. *hansenii* strains showed different abilities to proliferate in media containing nitrate and nitrite and that both substances affected the production of volatile compounds [86]. Later on, the effects of reducing nitrate and nitrite on microbial growth and aroma production during the ripening time were determined in more detail [87]. Nitrite and nitrate reduction in fermented sausages did not affect microbial growth but decreased lipid oxidation and the generation of derived volatiles. *D. hansenii* inoculation limited lipid oxidation and increased the generation of volatile compounds derived from amino acid degradation and esterase activity, and the antioxidant capacity of *D. hansenii* during the drying time prevented nitrite oxidation. It was proposed that, in summary, yeast inoculation counteracts the negative impacts of nitrite and nitrate reduction on aroma due to its antioxidant capacity during drying time, aroma production, and hindering of nitrite oxidation.

Ongoing experiments in the laboratory of the authors provide results that agree with those commented above. In this case, the inoculation of loin pork with a selected strain of *D. hansenii* may allow a reduction in the amounts of preservatives (nitrite/nitrate/salt) used during the applied manufacturing process (not published).

### 5.2. Biocontrol of Toxigenic Moulds

The environmental conditions during the ripening of meat products favour the growth of toxigenic moulds on their surface. Some of these moulds can produce mycotoxins on such meat products. Mycotoxins are secondary fungal metabolites that cause diseases affecting the immune system, nervous system, liver, kidneys, blood, and some mycotoxins are known to be carcinogens [88]. Biocontrol by antagonistic microorganisms has been proposed for controlling toxigenic moulds in foodstuffs. Several research groups have recently focused on the study of the potential role of *D. hansenii* to control growth and/or the production of mycotoxins by toxigenic moulds in order to minimize this hazard.

**Figure 2:**
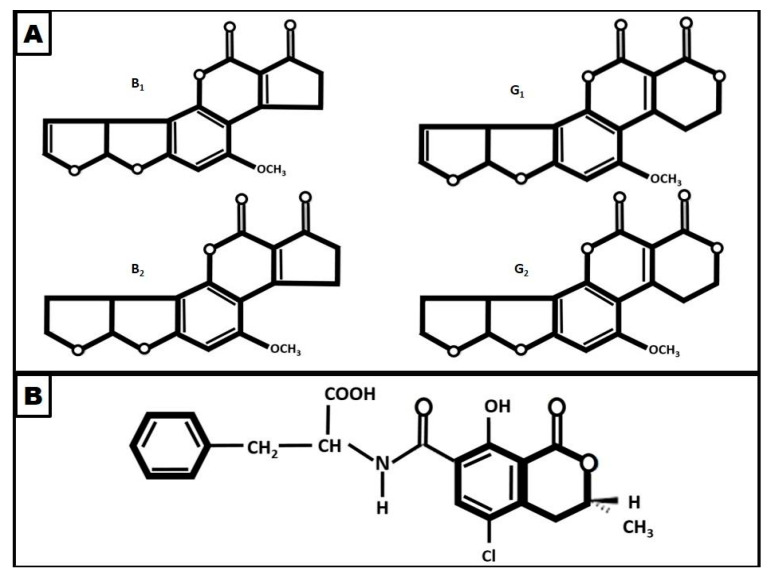
Chemical structure of the mayor mycotoxins found in dry-cured meat products: aflatoxins B1, B2, G1, and G2 (**A**); and ochratoxin A (**B**).

Aflatoxins (AFs) and ochratoxin A (OTA) are the two major mycotoxins found in dry-cured meat products (Figure 2). While AFs, produced by several species of *Aspergillus* such as *A. flavus* or *A. parasiticus*, are considered the most toxic, OTA, produced by some *Aspergillus* and *Penicillium* species, is the most frequently encountered mycotoxin in dry-cured meat products, thus their study and control are of fundamental importance.

The mechanisms of action in antifungal yeasts are diverse: (i) competition in the ecosystem [53,89,90,91]; (ii) production of volatile compounds with antifungal activity [92,93,94,95]; and (iii) killer proteins or glycoproteins named mycocins (also known as killer toxins). Mycocins are defined as extracellular proteins with different activities, reportedly with an important main mechanism of action being the inhibition of β-glucan synthesis in the cell wall of sensitive microorganisms [96,97,98]. In this respect, once again, the heterogeneity of *D. hansenii* is very wide [95], however many strains show an antimould effect that does not negatively affect the general characteristics of meat products [51,99]. Relatively abundant information in relation to *D. hansenii* and OTA is available. *D. hansenii* can inhibit growth of two ochratoxigenic fungi, *P. nordicum* and *P. verrucosum*. Additionally, a decrease in OTA production and accumulation in food due to the presence of *D. hansenii* has been reported [90,91,95,100]. Very recently, the effect of *D. hansenii* on *A. westerdijkiae* ochratoxin A production and growth has been also described [101], however, it is important to mention that when a starter culture containing bacteria plus *D. hansenii* was co-inoculated with *A. westerdijkiae* or *P. nordicum* in meat-based media, OTA production was stimulated indicating possible unforeseen safety problems [102]. These findings are an important wake-up call regarding the need for specific studies in each case and, in fact, very recently new information indicating that *D. hansenii* reduced the OTA levels produced by *P. nordicum* in dry-meat products has been provided [99].

Much less information has been published in relation to the potential use of *D. hansenii* selected strains on aflatoxins production. However, the combination of *D. hansenii* and the small antifungal protein, PgAFP, or the use of single native *D. hansenii* strains have been proposed to inhibit the growth of *A. parasiticus* and reduce aflatoxin production in meat products [103,104].

As already mentioned, the mechanisms of action in antifungal yeasts is varied. The effect of antifungal yeasts on the reduction of mycotoxin biosynthesis at the transcriptional level has warranted several studies, although much more research is needed in order to achieve a global view of the field. A repression of the expression of genes involved in OTA biosynthesis has been reported in *Aspergillus* and *Penicillium* species as consequence of the presence of *D. hansenii* on yeast-modified Czapek-Dox agar [105], on meat model systems [100], and in dry-cured ham [106]. Therefore, studies related to the effect of yeasts on the gene expression involved in the biosynthetic pathways of mycotoxins would be of utmost interest. This is one of the crucial aims for the future and further investigations focused on the understanding of the different modes of yeast antifungal actions are necessary to enhance and predict the effect of protective cultures of *D. hansenii* on sausages and dry-cured meat products.

Finally, it is worth mentioning that the efficacy of potential biocontrol agents is influenced by both the nutritional sources and inherent environmental factors of the dry-cured meat product processing. The halotolerant character of *D. hansenii* [17,18] makes its application as bio-preservative in dry-cured meat products very adequate due to their salt content. Consequently, it would be interesting to identify the environmental conditions in which the biocontrol agents have the highest effectiveness against the common toxigenic moulds found in these products. In fact, our group has shown that the presence of sodium in the external medium improves the performance of *D. hansenii* against different abiotic stresses [58,107,108], and several studies have reports the positive effects of the presence of NaCl during the use of *D. hansenii* as a biocontrol agent. On the one hand, yeast inhibitory activity of *P. nordicum* and OTA production was enhanced by the presence of NaCl [90], and on the other hand a killer toxin produced by *D. hansenii* showed optimum inhibitory effects against different unwanted microorganisms in the presence of 8% NaCl [109].

## 6. Conclusions and Perspectives

**Figure 3:**
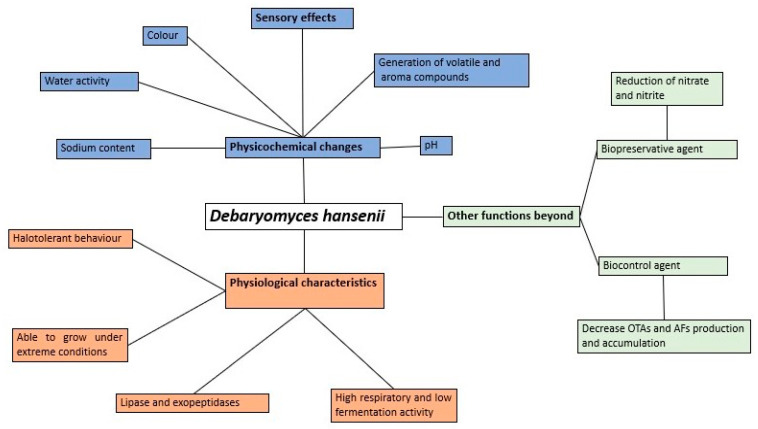
Defining the biological relevance of *Debaryomyces hansenii* on the final characteristics of sausages and dry-meat products.

*Debaryomyces hansenii* is, most probably, the most abundant yeast found in sausages and dry-meat products manufactured all around the world. However, its specific role on the final characteristics of the products has been controversial. It could be considered a necessary organism or a mere friendly guest. Figure 3 summarizes the multiple consequences that the presence of *D. hansenii* may have on the characteristics of the products. The diversity of meat products, the heterogeneity of the strains, or the multiplicity of manufacturing processes makes impossible to predict what the final result would be after the inoculation of a given meat product with a selected *D. hansenii* strain, at a certain concentration, and under specific conditions, without a preliminary detailed study according to what the industry needs and the consumer demands. From this point of view, it is important to highlight the use of terroir strains and indigenous microbial communities to potentially improve and keep the properties and quality of the products [110].

What is very conclusive is that there are no reports informing of unwanted negative effects or even health problems due to the inoculation of meat products with *D. hansenii* and, in the worst scenario, the presence of the yeast does not affect the overall quality and typical features of the products.

Globally speaking, the effects of *D. hansenii* on pH, water activity, moisture, or lipid composition are not especially relevant. However, *D. hansenii* increases carbohydrate fermentation and amino acid catabolism and modifies the levels of volatile and aromatic compounds: it increases the generation of esters such as ethyl esters or alcohol compounds, and affects sulphur production. Consequently, there is a tendency to agree that the use of selected strains has a positive effect on final flavour, on the sensory characteristics and, in many cases, on consumer acceptance.

There are important new aspects of research in the field beyond simply improving the general sensory characteristics of sausages and dry-meat products. The contribution of *D. hansenii* on the safety of inoculated sausages and dry-meat products is a subject to be developed in the near future. Global integration and understanding of *D. hansenii* metabolic activities are needed. This yeast is potentially suitable as a bio-preservative agent. *D. hansenii* degrades biogenic amines and its presence may contribute to decreasing the use of some preservatives and strains that inhibit mycotoxins production and unwanted moulds growth.

In conclusion, our knowledge regarding the possibilities of using *D. hansenii* in the manufacturing of meat products is quite extensive, but far from complete. New advances in the study of yeast physiology and molecular biology are likely to bring new insight with both expected and novel impacts in the future. In this sense, the investigation of this yeast as a biocontrol agent or as a tool to obtain more ecological meat products will provide findings directly applicable to the food industry.

## Figures and Tables

**Table 1:** A selection of sausages and dry-meat products from which *Debaryomyces hansenii* has been isolated.

Product	Origin	Brief Description	References
“Cacholeira”	Portugal	Traditional Portuguese sausage with delicate flavour obtained from the offal and fat of the pig.	[44]
“Chorizo”	Spain	Traditional Spanish cured meat product made from coarsely chopped pork and pork fat seasoned with garlic, “pimentón”, and salt.	[5]
“Jamón ibérico”	Spain	High-quality variety of “jamón” produced in Spain and Portugal.	[37,40]
“Jamón”	Spain	Meat product from pork typical of Spanish cuisine	[48]
“Lacón”	Spain	Cured meat product obtained from the shoulders or front legs of the pig.	[49]
“Salame di senise”	Italy	Traditional dry sausage from the Sinni Valley in the Basilicata region.	[38]
“Salchichón”	Spain	Traditional Spanish cured meat generally made of pig, although other meats can be used.	[29,50]
“Salsicca sarda”	Italy	Traditional fermented dry-cured sausage produced exclusively in Sardinia.	[51]
“Soppressata of Vallo di Diano”	Italy	Traditional Southern Italian dry-fermented sausage.	[52]
“Sucuck”	Turkey	Semi-dry, spicy Middle Eastern sausage with a high fat content traditionally prepared with ground beef and spices.	[39]
Dry-cured Parma ham	Italy	Famous variety of “prosciutto” from the Parma region in Italy.	[53]
Fermented sausage	Norway; Denmark; Italy; United Kingdom; Spain	Diverse kind of fermented meat.	[8,13,42,54,55]
Greek dry salami	Greece	Traditional Greek dry-cured meat.	[36]
Laowo dry-cured ham	China	Traditional Chinese dry-cured ham obtained from the hind leg of the pig.	[46]
Llama meat sausage	Argentina	Traditional products consumed in the Andrea region of South America.	[45]
Mianning ham	China	Traditional fermented meat product in Meanning, characterized by the use of plump muscle and small legs.	[10]
Panxian ham	China	Famous dry-cured ham in China characterized by strong taste, flavour, aroma, and texture.	[47]
Pork loin	Spain	Cured meat product prepared by removing fat from pork followed by seasoning for six months.	[56,57,58]
Vienna sausage	Austria	Thin, parboiled sausage traditionally made of pork and beef in a casing of sheep’s intestine.	[7]

**Table 2:** Main factors influencing the effect of *Debaryomyces hansenii* on the final characteristics of sausages and dry-meat products.

Factor	Comment
Strain	Very heterogeneous behaviour
Cell amount inoculated	The concentration of ufc/g of product greatly varies in the literature
Form of application	Spread on the surface or mixed with the rest of components of the product
Presence of additional microbial starters	Presence of bacterial and/or fungal starters
Meat product	Acutely diverse meat products
Manufacturing conditions	Time and temperature of ripening, presence of spices, etc
